# Vocal Communications and the Maintenance of Population Specific Songs in a Contact Zone

**DOI:** 10.1371/journal.pone.0035257

**Published:** 2012-05-04

**Authors:** Jonathan T. Rowell, Maria R. Servedio

**Affiliations:** Department of Biology, University of North Carolina at Chapel Hill, Chapel Hill, North Carolina, United States of America; Claremont Colleges, United States of America

## Abstract

Bird song has been hypothesized to play a role in several important aspects of the biology of songbirds, including the generation of taxonomic diversity by speciation; however, the role that song plays in speciation within this group may be dependent upon the ability of populations to maintain population specific songs or calls in the face of gene flow and external cultural influences. Here, in an exploratory study, we construct a spatially explicit model of population movement to examine the consequences of secondary contact of populations singing distinct songs. We concentrate on two broad questions: 1) will population specific songs be maintained in a contact zone or will they be replaced by shared song, and 2) what spatial patterns in the distribution of songs may result from contact? We examine the effects of multiple factors including song-based mating preferences and movement probabilities, oblique versus paternal learning of song, and both cultural and genetic mutations. We find a variety of conditions under which population specific songs can be maintained, particularly when females have preferences for their population specific songs, and we document many distinct patterns of song distribution within the contact zone, including clines, banding, and mosaics.

## Introduction

Vocalization is one means by which communication occurs between conspecifics and/or heterospecifics, and it can transmit identification, threat alerts [1], territorial claims or attraction of potential mates [2], [3] or need advertisement [4], among other uses. The set of signals associated with any given system is derived through some combination of genetically-linked factors (e.g. morphological and neurological development) and possibly socio-cultural learning processes. One noted example of vocal communication is the repertoire of songs and calls produced by birds. Among the songbirds (Oscines, order Passeriformes, suborder Passeri), which learn their songs, a single species may consist of distinct populations singing different song variants, e.g. [5]–[7]. Similarly, learned calls may also vary between populations in songbirds (e.g., [8]) and even some non-passerine birds, such as parrots (Order Psittaciformes, e.g., [9]). The existence of distinct vocal dialects, or song traditions shared by local birds, may affect several aspects of the behavioral ecology of birds including mating success, territory formation, and potentially movement (reviewed in [10], [11]). Both empirical (e.g., [12], [13]) and theoretical research (e.g., [14]–[18]) have previously been used to address the formation and evolutionary effects of songs and dialects. In this paper, we focus on a theoretical study and numerical simulation of the interaction of two genetically-distinct populations or incipient species across a network of local sites when these groups possess partially overlapping communication options (e.g. songs or calls) that affect mate selection and dispersal behavior. In particular we conduct an exploratory study of patterns of song maintenance between two such groups in a contact zone, by developing a spatially explicit, deterministic population model in which the divergence between the two groups in song production and, in some cases, female preference are partially genetically based. Within the contact zone, this genetic predisposition may result in partial reproductive isolation via mate choice; thus these groups are perhaps best considered “regiolects” *sensu* Martens [19]. For our purposes here, we consider an intermediate spatial scale wherein stochastic effects on local population levels (or densities) are ignored but observation of adjacent sites remains possible.

We are particularly interested in observing whether distinct, population specific songs or calls can be maintained following secondary contact. The exact forces and conditions necessary for the maintenance of song variation have been the subject of much study and debate (reviewed in [11]). It has been hypothesized that female preferences may play an important role ([20]). Consider that many species can differentiate between conspecific and heterospecific songs (see, e.g., references in [21]), even when these songs are culturally transmitted rather than genetically inherited (e.g. [21]–[25]). Similarly, in several species with dialects, females have been shown to prefer local dialects over foreign ones ([26]–[32] but see e.g. [33], [34]). If assortative mating based on song were of sufficient strength, it could restrict gene flow, potentially leading to speciation or further divergence between species (see discussion in [10]).

The role of dispersal in the maintenance of variation in song is less clear, and it may be that different mechanisms are operating in different species to maintain this variation when dispersal is not restricted by physical barriers between groups. In some cases birds learn local songs or calls after dispersing into a group (e.g., [35]–[37]). In other cases, juveniles learn a greater number of songs than are typically performed by adults, but they subsequently narrow their repertoire to match local dialects after dispersal through selective attrition (e.g., [38]). Finally, Marler and Tamura [5] suggested males preferentially disperse to areas where the local males already sing familiar song (see also [39]). Consistent with this last suggestion, several studies found evidence that suggests that dispersal may be reduced across dialect boundaries [40]–[42], while others simply document a correlation between dialect boundaries and genetic differences or reductions in gene flow between populations (e.g. white-crowned sparrow [43]–[45]. Correlative studies like this, however, can not distinguish whether the lack of dispersal allows the formation of distinct dialects or regiolects (e.g. [46]) or vice versa (males with unfamiliar song may have reduced success at establishing and maintaining territories due to an inability to communicate with male competitors (e.g. [43], [47]–[49]).

Yet other studies counter the suggestion that dialects would lead to substantial genetic divergence (e.g., [50]–[55]). In fact the mechanism of dialect maintenance via song-based dispersal has largely fallen out of favor in recent years. This remains an understudied area [11], however, and it is possible that dispersal based on dialect may be important in some species. Because of this uncertainty, we examine the effects of both song-biased and song-neutral dispersal on the maintenance of song variation within our model.

The initial establishment of a novel dialect or communication group is itself an intriguing question. Thielcke [56] hypothesized that groups of young birds, without a fully learned adult song, might colonize new areas and establish a novel dialect together (see also Baker [57]), and Mundinger [20] found evidence consistent with the rapid formation of dialects (∼20 years) via this type of mechanism in a colonization front of house finches. Additionally, Slater and colleagues (17,18) showed via computer simulations that dialects in both very local and larger neighborhoods can form within a single population depending upon the distribution of song types and copying rules. Our own model is presented in the context of regiolects formed in allopatry followed subsequently by secondary contact between divergent populations. One song type might become predominant, the song types could merge resulting in the formation of mixed song (e.g., [24], [39]), or songs from both regiolects could be maintained in some spatial pattern (perhaps analogous to either a cline or a mosaic hybrid zone).

In this exploratory study, we use a spatially explicit model to simulate the potential outcomes of secondary contact between well-established populations with distinct genetic predispositions and initially producing exclusive songs. This contact could be between regiolects formed in allopatry or between ones generated in sympatry or parapatry with a subsequent disruption in gene flow, e.g. recolonization in a patchy environment. We assume that these populations are well enough established to have involved a genetic shift in a neurological template for song recognition. We are interested in the following questions: When there is secondary contact between the populations, can differences in song be maintained, or will contact zones instead lead to the predominance of mixed songs or songs shared between the populations? If population specific songs are maintained, do they form distinct and predictable patterns within the contact zone? We find the maintenance of song variation where songs occur in a variety of spatial patterns, and assess the dependence of this maintenance on a variety of factors.

## Methods

To study the outcome of secondary contact between two vocal communication systems – here represented as bird populations singing potentially distinct sets of songs – we developed a simple discrete-time, deterministic simulation model (detailed in Appendix S1) of two regional, genetically distinct populations with overlapping generations expanding into a spatially organized network of patches defined as a rectangular lattice 25 patches long (x) and 10 patches wide (y). Each patch, which can be thought of as a local neighborhood in which both resource competition and song learning can occur, is assumed to contain the same level of resources and is thus ultimately capable of supporting the same total population density. Ellers and Slabbekoorn [14] also study dialects using a spatially structured model, in their case of territories on a grid, but they were considering dialect formation within a single populated region, not the colonization of a new area during secondary contact (and hence the maintenance, not generation, of song variation). Conversely, Olofsson and Servedio [15] considered the situation of secondary contact, but did not include a spatial component to their model, which found that when the degree of learning was allowed to evolve song variation tended to be lost. Dispersal rules are associated with recent models of adaptive movement across heterogeneous landscapes (e.g. [58]–[60]) and incorporate considerations of both resource and socio-cultural benefit.

We assume that the two populations of birds have diverged prior to their introduction to the contact zone such that there has been a change or shift in the underlying template for song production and recognition (e.g. [61]). This shift is incomplete in that there is some overlap in the songs allowed by each population’s template. The song structure in this model is based roughly on the models of Lachlan and colleagues [61]–[63]. Model individuals are haploid and are categorized by their phenogenotype, i.e. the combination of an allele coding for their inherent song template and of the specific song actually produced (males) or preferred (females). Specifically, we assume that there are a total of 6 different songs, which are well-ordered according to their similarity to one another (i.e. 1 is most similar to 2, then 3, etc.). We assume that the template associated with the allele *A* at locus **A** (fixed in population 1), allows recognition and performance of songs 1–4 (producing phenogenotypes *A*1–*A*4), while allele *a,* which is fixed in population 2, allows recognition of songs 3–6 (producing phenogenotypes *a*3–*a*6). Songs 1 and 2 are exclusive to population 1, while songs 5 and 6 are restricted to population 2. Songs 3 and 4 are either shared or mixed songs potentially sung in both populations. This gives a total of 8 possible phenogenotypes (*A*1–*A*4, *a*3–*a*6).

As a modeling simplification, the use of haploidy rather than diploidy is a common assumption of population-genetic models–particularly trial or exploratory studies such as this. As a necessary caveat, our results do not preclude alternative dynamics ensuing from diploidy–questions of hybrid vigor or sterility, dominant, recessive or epigenetic trait expression, etc. all become potentially interesting directions of research. Rather they provide a baseline from which we can then compare future research extensions.

The model life cycle is comprised of four distinct steps: parental mating and the generation of newly hatched offspring, offspring learning a specific song or mating preference, the dispersal of young adults into directly neighboring patches, and an annual mortality event. During mating, adult females preferentially select only males singing songs recognized by their template [64]–[67]: specific mating preference weights *a_ij_* are given by a set of affinity scores (see “Affinity Schemes” below). Genetic mutation in offspring (γ_2_) is an optional feature of the model at this stage. This may also approximate to rare density-dependent long-distance dispersal of naïve foreign young from outside of the contact zone. Next, based upon the song recognition template controlled by the genotype, learning occurs in which males develop songs (production learning) and females develop mating preferences for songs (perceptual learning). We thus assume that songs heard early in development influence perception and preferences in both sexes in complementary ways from the same genetic basis (e.g., [68]). Template-recognizable songs serving as models for learning are typically selected in proportion to their relative frequency within the local patch community (oblique cultural transmission); however, paternal transmission of song can occur with non-trivial probability *p*. We also include the possibility of error during transmission (cultural mutation, γ_1_). Dispersal then occurs, in which young adults disperse based upon resource availability (resource movement with sensitivity ω_1_), song similarity (cultural movement with sensitivity ω_2_), and/or random movement (diffusion, ω_3_). When song similarity is involved in movement, individuals move towards patches with significant representation of familiar song, based again on the weightings described in “Affinity Schemes”, and away from patches with predominantly unfamiliar song. Finally, a density-related per capita mortality on all adults is applied at the end of each generation.

### Affinity Schemes

**Figure 1:**
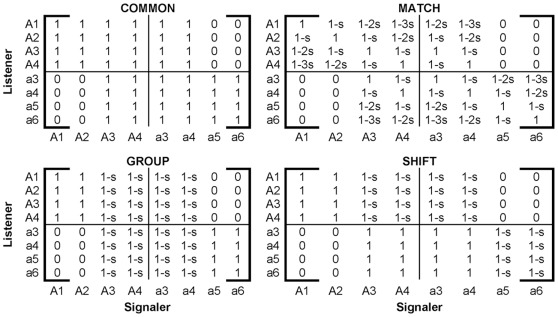
Affinity schemes. The matrices represent the relative weight that a listener of phenogenotype *i* gives to a singer of phenogenotype *j* for each of the four affinity schemes. The phenogenotypes of listeners and receivers are labeled on the schemes. Break lines have been included to group cases by genotype. The variable *s* describes a reduction in the affinity. Default values of *s* are: Match, *s* = .1; Group or Shift, *s* = .25. The Common scheme is equivalent to any of the above schemes with *s* = 0.

There are four primary affinity schemes used in this model: Common, Group, Shift, and Match. Each is represented by an 8-by-8 matrix (Fig. 1) in which the entry *a_ij_* is the affinity that a receiver of phenogenotype *i* has for a signaler of type *j*. The Common Scheme, which can be construed as a base model from which all other schemes diverge, can be obtained using any of the other three affinity schemes and setting the non-preferred discounting to zero, *s* = 0. Shared songs receive the same affinity independent of the genotype of the singer (i.e. *A3* and *a3* are treated equally, as are *A4* and *a4*). Songs specific to the opposite population are scored with 0 affinity. Note that except for the Match affinity scheme, receivers who share a common allele are functionally equivalent during mating or movement regardless of phenotypic category.

Under the *Common* Scheme, all recognized songs (i.e. those which would be naturally produced by the listener’s genotype) receive equal affinity, *a_ij_* = 1, while foreign songs are scored 0. Under the *Group* Scheme, there is an inherent preference for songs specific to one’s genetic population (*a_ij_* = 1) over songs shared between the populations (*a_ij_* = 1-s) regardless of the listener’s formal phenotype. Under the *Shift* Scheme, both genotypes favor producible lower indexed songs over higher indexed songs (perhaps as a result of sensory bias, or differences between higher and lower quality songs for which the index is a proxy), but they appear to have “shifted the window” of preference in song-space relative to one another. Thus *A*-allele individuals exhibit an innate bias towards their specific songs (perhaps novel songs developed during isolation), while *a*-allele individuals favor shared songs 3 and 4 over those specific to its genotype (5 and 6). With the final affinity scheme, *Match*, one’s affinity is strongest for songs that match the learned song type (affinity *a_ii_ = *1). Affinity for recognizable, but less preferred, songs diminishes by *s* for each song step away from the matching ideal (*a_ij_* = 1−s*|*i–j*|).

### Simulation Protocol

At the start of each simulation, population 1 is fixed for the phenogenotype *A*1, which is present with a uniform density along the edge of the grid defined by *x* = 1 (1≤*y*≤10). Population 2 is similarly fixed for *a*6 and present at *x* = 25 (1≤*y*≤10). All other locations within the grid are initially empty. The operative sex ratio in the model is 1, and we will only track density for a single sex.

To approximate the state of the population at equilibrium, simulations were run in batches for 2000 generations, a period of time noted sufficient for absolute changes in local phenogenotype densities per time step to drop below 5×10^−3^ (or approximately.01% per annum change relative to maximum population size). Except in certain instances of relatively high cultural sensitivity (noted in Results), all patches were colonized and reached a total local population density equal or near to carrying capacity. Unless noted otherwise, for each simulation batch listed below, resource and cultural movement sensitivities, ω_1_ and ω_2_, were varied over five orders of magnitude to the values 10^−6^, 10^−5^, 10^−4^, 10^−3^, and 10^−2^, using all twenty-five parametric combinations. Paternal imprinting on offspring was ignored in the initial simulations (*p = 0*). See the Appendix S1 for additional standard conditions and code implementation.

#### The simple model and diffusion

In these simulations, we examined the patterns of development within the contact zone for the basic model without either cultural or genetic mutation and without paternal imprinting. Diffusion was introduced at different levels (ω_3_ = 0, 10^−8^, 10^−4^, and 10^−2^). These simulations were done first under the Common scheme and then for each of the three remaining primary affinity schemes.

#### Cultural and genetic mutation simulations

Simulations were performed that added either cultural mutation (γ_1_ = .10, .05, .01, and.001) or genetic mutation (γ_2_ = 10^−6^, 10^−4^) – but not both –to the simple and diffusive models above. Additional simulations incorporated both cultural and genetic mutation in the diffusion model only.

#### Variation in paternal transmission

The simulations described above were performed again, but here we developed a secondary feature of the model: paternal transmission during the song learning stage of the life cycle. We varied the frequency of offspring (*p* = 0, .1, ….9, 1.0) deriving their phenotype from that of their sire.

#### No female preference

In addition to the four primary schemes above, we also consider three schemes that are “mixed” in the sense that different affinity scores are used for female preference and for movement. Specifically, we assumed that there is no female preference in mating between recognized song types (*s* = 0, Common). Movement was alternatively governed under the Group, Shift, or Match affinity scheme.

#### Spatial variations

In our final simulations, we spatially varied the initial densities of the *A*1 and/or *a*6 phenogenotypes along their respective edges of the grid (mean density 10, standard deviation 2.5) to determine whether non-uniformity in the initial colonizing front could result in large-scale clustering of phenogenotypes.

## Results

**Figure 2:**
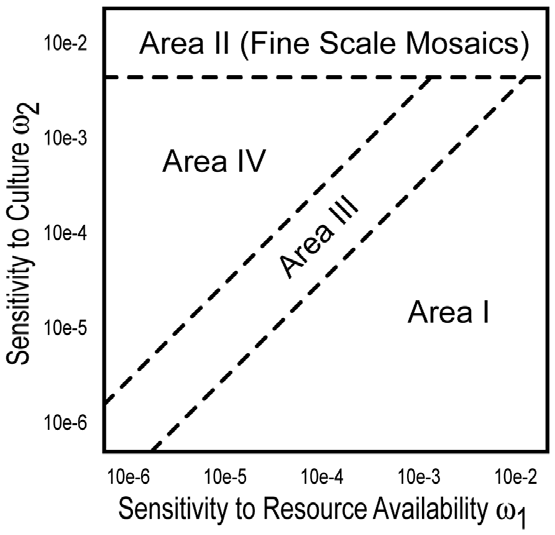
Parametric Regions. The schematic drawing indicates areas in parametric space (ω_1_, ω_2_) that tend to produce similar outcomes. Area I corresponds to the default scenario where sensitivity to resource availability exceeds that of sensitivity to song similarity. Area II is a phenomenological threshold where fine scale mosaics develop. Area III corresponds to when there is a comparable emphasis on resource and cultural needs during movement. Area IV represents parameter combinations with a greater emphasis on cultural needs during movement but not producing fine scale mosaics.

Our simulations lead to a variety of possible outcomes for the relative prevalence and spatial distribution of phenogenotypes. Several of the patterns we have identified occur under restricted parameter combinations, and Figure 2 diagrams the major parametric “areas” for combinations of our two primary parameters, resource and cultural sensitivity. In Area I, movement within the contact zone is relatively more sensitive to resource availability than cultural (songtype) composition of local sites. This is expected to be the most commonly observed scenario – in particular, the limiting case of no cultural sensitivity during movement is thought to be most prevalent. Area II is defined by passing a threshold limit of cultural sensitivity, and it is consistently characterized by a fine scale mosaic distribution (except with the basic simple model) in which the spatial correlation of phenogenotypes is highly variable. Examples include landscapes of embedded, isolated phenogenotypic groups. These mosaics are a natural consequence of spatial instability induced by acute sensitivity to cultural factors, regardless of resource concerns. Resource and cultural sensitivities are of comparable magnitudes in Area III, while Area IV contains parameter combinations whose cultural sensitivity is relatively higher but below the mosaic threshold of Area II. Area IV is distinct in that it has been observed to admit multiple pattern types for some trials, suggesting finer subdivisions of the parameter space below the resolution of the simulations.

**Figure 3:**
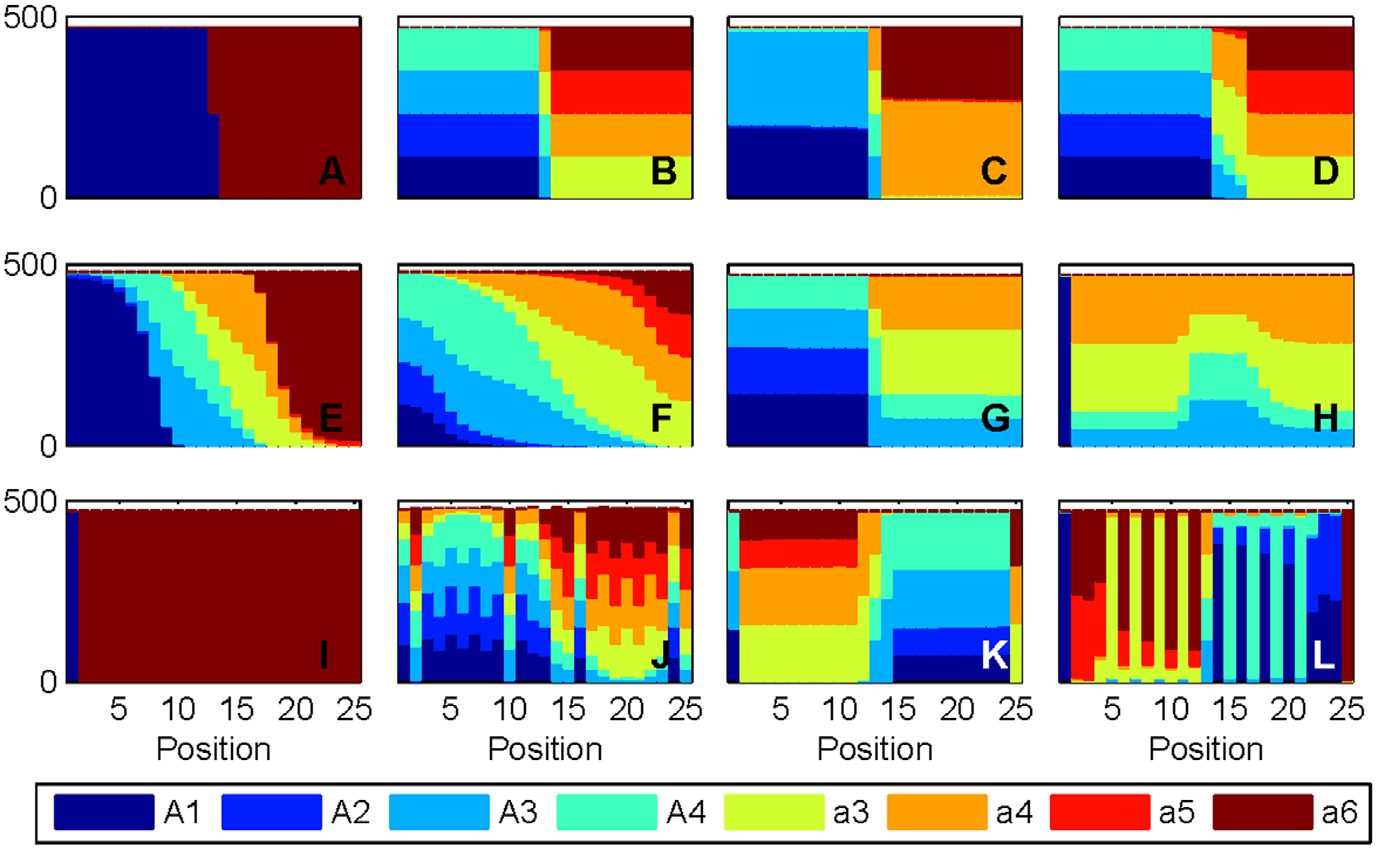
Representative sampling of different model outcomes. Each panel represents a stacked bar plot showing transect population totals (summed across *y*) by phenogenotype at position *x*. Distributions are uniform across transects (independent of position *y*) except for fine scale mosaics (3J). Parameters other than ω_1_ or ω_2_ are 0 unless otherwise specified. Scheme abbreviations: (Co)mmon, (Gr)oup, (M)atch, (Sh)ift. A) Area I, Co; B) Area I, M, γ_1_ = 5%; C) Area I, Co, γ_2_ = 10^−6^, γ_1_ = .01%; D) Area I, Sh, γ_1_ = 5%; E) Area I, Gr, γ_1_ = .01%, ω_3_ = 10^−2^; F) Area I, M, γ_2_ = 10^−6^, γ_1_ = .5%, ω_3_ = 10^−2^; G) Area I, Sh, γ_2_ = 10^−6^, γ_1_ = 1%; H) Area III, Sh, γ_2_ = 10^−4^; I) Area III, Sh; 3J) Area II, M, ω_3_ = 10^−2^, γ_1_ = 5%; 3K) Area III, Co, γ_2_ = 10^−6^; 3L) Area IV, M, γ_2_ = 10^−6^.

Simulation outcomes can be generically assigned to handful of categorical patterns (Figure 3); however, we note that even within these broadly defined patterns there is a degree of variation in relative phenogenotypic frequencies. We also caution that these patterns are not necessarily exhaustive and that other outcomes could have fallen unobserved between our selected parametric combinations. Patterns include: 1) an even division of the contact zone by genotype with or without a central zone of mixed song phenogenotypes (Fig. 3A–D); 2) a gradient distribution of genotypes (Fig. 3E,F); 3) an even division of the contact zone between one genotype vs. assorted mixed songs (Fig. 3G); and 4) widespread occurrence of mixed songs (Fig. 3H). Furthermore, relatively high cultural sensitivity (Areas II-IV of Fig. 2) support: 5) either an empty interior or expansion of a single genotype (Area III–IV, e.g.,Fig. 3I); 6) fine scale mosaics in which the spatial auto-correlation of a given phenogenotype varies significantly with patch distance (Area II, Fig. 3J); 7) inversions in which genotypes occupy the half of the contact zone further from their introduction (Area III, Fig. 3K); and 8) banding or broad alternating patterns of genotypes, shared vs. exclusive songs, or even between exclusive songs (Area IV, Fig. 3L).

### Basic Simple Model

We begin by describing a basic simple model without diffusion, mutation, or paternal transmission and which operates under the Common affinity scheme for both mating and movement (i.e. *s* = 0 for any of the affinity schemes). All parameters except ω_1_ and ω_2_ are set to 0. When resource availability is the stronger factor in movement (ω_1_>ω_2_, Area I), phenogenotypes *A*1 and *a*6 evenly divide the territory (Figure 3A). When the desire for song similarity is comparable to or stronger than resource needs for movement (ω_2_≥ω_1_, Areas III–IV), however, neither population expands beyond the sites of their original introduction. This peculiar result is related to the initial population sizes being sufficiently large to offer a cultural attractant that prevents expansion. This basic finding is replicated for all affinity schemes, except that under Shift, the initial *a*6 phenogenotype population size is not sufficiently auto-attractive when ω_1_≈ω_2_ (Area III) to arrest expansion (e.g. Figure 3I).

### Cultural Mutation (Figs. 4 and 5, Columns 1–2)

**Figure 4:**
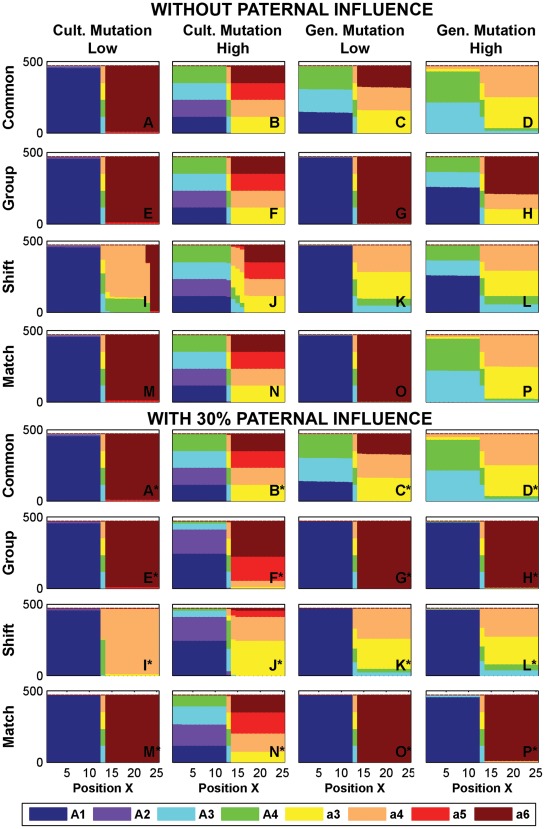
Area I under cultural or genetic mutations. Panels here are as those described in Figure 3. ω_1_ = 10^−4^, ω_2_ = 10^−6^ in all panels. Rows correspond to the affinity schemes Common, Group, Shift, and Match, respectively. Column 1: γ_1_ = .01%; Column 2: γ_1_ = 5%; Column 3: γ_2_ = 10^−6^; and Column 4: γ_2_ = 10^−4^. All other parameters assumed 0. Panels A*–P* show the effect of adding 30% paternal transmission to learning.

**Figure 5:**
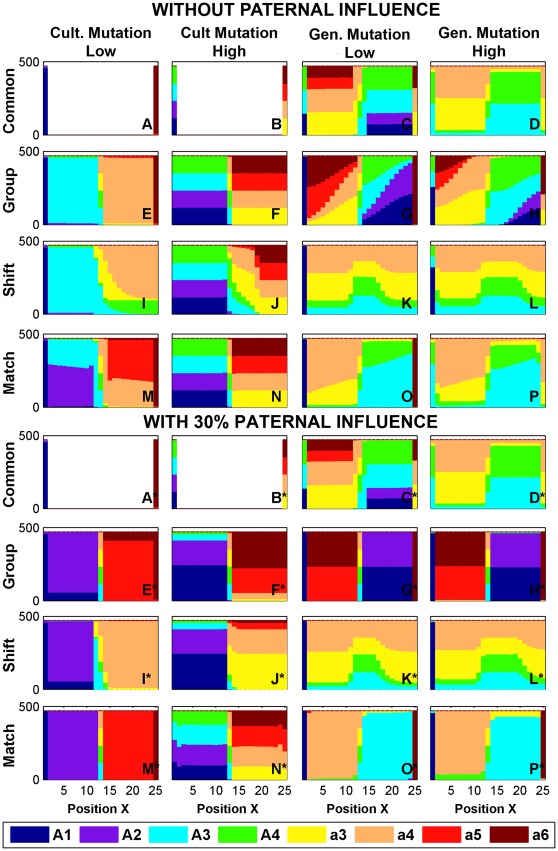
Area III under cultural or genetic mutations. Plots are as in Figure 4, but with parameters ω_1_ = 10^−4^, ω_2_ = 10^−4^.

Cultural mutation has the effect of introducing non-original phenogenotypes to the contact zone. In Area I, low levels of cultural mutation (γ_1_ = .01% to.1%, Fig. 4, column 1) create an intermediate contact barrier (1–5 patches wide, although it is exceptionally wider under Shift) between zones dominated by the original *A*1 and *a*6 phenogenotypes (e.g. Fig. 4A) and which is populated primarily by singers of shared song. When culture motivates dispersal to a comparable or greater extent than resource needs (ω_2_≥ω_1_, Areas III–IV, Fig. 5), non-original phenogenotypes become widespread at low cultural mutation levels (e.g. *A*2 or *A*3, Fig. 5E, I, M) due to their relatively greater contributions to colonizing fronts. At higher levels of cultural mutation (1%–5%, column 2 of Figures 4 and 5), the original exclusive song types are no longer dominant, and all songs within a genetic predisposition are comparably represented in their respective halves of the contact zone. [Note, “low” and “high” rates of mutation are phenomenological distinctions based upon common simulation results. Some estimates of actual cultural mutation rates are well above 1–5% [65], but the corresponding simulation outcome is categorically similar to those with 1–5% mutation rates. Moreover, we have found no empirical estimates of mutation rates below 1%.].

### Genetic Mutation (Figs. 4 and 5, Columns 3–4)

Genetic mutation had the greatest immediate effect on the maintenance of exclusive songs. At low mutation levels (γ_2_ = 10^−6^) under the Common affinity scheme, the original *A*1 and *a*6 phenogenotypes are reduced in favor of shared songs in Area I (Figure 4C), and they are effectively removed at high mutation levels (γ_2_ = 10^−4^, Figure 4D). The shared songs are abundant because singers of one genotype share their song with similar singers within the other genotype. This artificially inflates their relative frequency with regard to the selection of role models during song learning. Genetic mutation also leads to other unique patterns – inversions (Area III; Figs. 3K and 5, columns 3,4) where each genotype is predominantly represented in the half-territory closest to the original inhabited locations of the other genotype, and banding (Area IV) where the inversion process has consistently repeated to generate alternating bands of phenogenotypic frequencies. The inversion or banding is produced by the colonization wave of initially rare mutants leaving the introduction sites and proceeding to expand (at low density) further into the center of the contact zone (the common phenogenotypes do not move as rapidly because of the strong cultural affinity). Only amid fine scale mosaics (area II) do exclusive songs appear in appreciable numbers.

Non-Common affinity schemes can mitigate the reduction in frequency suffered by the original exclusive songs *A*1 and *a*6 within Area I when genetic mutation is low (Fig. 4G,K,O). Even at high genetic mutation, exclusive phenogenotypes that are favored (i.e. under Group or Shift) still constitute a plurality. Shift is notable in that the results are asymmetric – phenogenotype *A*1, which is favored by allele *A*, is supported while phenogenotype *a*6, which is not favored by allele *a*, is not. Inversions still hold in Area III (Fig. 5, except for Shift), but the support of exclusive songs is absent save by the Group affinity scheme. Finally at high γ_2_ in Area IV, exclusive songs can be seen in isolated or limited bands within the contact zone as one approaches the parametric limits of Area II (not shown).

### Cultural Mutation and Genetic Mutation

If both forms of mutation are included in the model, the observed patterns are primarily drawn from those seen with either cultural or genetic mutation alone. For the purposes of comparisons within this model, cultural and genetic mutation may be regarded as either high (γ_1_ = 5%, γ_2_ = 10^−4^) or low (γ_1_ = .01%, γ_2_ = 10^−6^) based upon qualitative differences in simulation outcomes. Typically when both rates are qualitatively low, the outcome resembles that which is observed with only low cultural mutation. When one mutation rate is high and the other is low, the outcome of the model will at least roughly resemble the results discussed above for whichever rate was high. Finally, when both rates are high, all phenotypes persist due to cultural mutation: Common and Match more closely approximate the results from high genetic mutation, while Group and Shift more closely resemble their results under the combination of high cultural mutation and low genetic mutation. Noted exceptions (Area I) to these trends include: 1) the Common scheme with γ_1_ and γ_2_ both low yields an even division of the contact zone with *A*1 and *A*3 paired against *a*4 and *a*6 (Fig. 3C); and 2) the Shift scheme with high γ_1_ and low γ_2_ yields an even territorial division between allele *A* (all songs present) and the collection of all mixed song phenogenotypes (Fig. 3G).

### Diffusion

The inclusion of diffusion in the Basic Simple Model and in models with mutation ensures that all locations within the contact zone are populated, and it blurs pattern distinctions between Areas I, III, and IV (Area II still produces fine scale mosaics, even without mutation, reflecting the natural spatial instability of the system). Genotype distributions change from an even, left-right partitioning of the contact zone to a frequency gradient derived from the results corresponding to Area I in the non-diffusive models (Fig. 3E,F). Additionally, patterns of predominantly shared song originally observed only very near the interface of the two populations now characterize more of the contact zone (e.g. Fig. 3E vs. 4E). The degree to which this intermediate zone spreads is predicated on the affinity scheme, with Group being the most restrictive and Common or Match the least.

### Paternal Transmission of Song (Figs. 4 and 5 Panels A* to P*)

Paternal transmission, with or without diffusion, has no meaningful effect without the concomitant addition of mutation to introduce alternate phenogenotypes. Moreover its effect depends greatly upon the affinity scheme used for mating. The Common mating scheme is largely unaffected by paternal transmission (Fig. 4 and 5, panels A* to D*) because there is no differential mating success between males of the same genotype at the same location, due to random mating within the genetic predisposition (*s* = 0). Paternal role models are thus selected in the same proportions as models during oblique transmission.

Paternal transmission does have an effect under the other affinity schemes. Consider that 1% cultural mutation (not shown) is a qualitatively high mutation rate that leads to equal levels of songs within a distribution (cf. Fig. 4N). Under Match, a mere 10% paternal transmission skews the local song distribution in favor of exclusive songs, and at 30%, the populations are almost entirely *A*1 or *a*6 (not shown). Even at 5% cultural mutation, a division of territory between the original exclusive songs *A*1 and *a*6 can be maintained for sufficiently high paternal transmission (50%, not shown). Group and Shift schemes respond to paternal transmission more clearly because of the innate preference for exclusive songs. Paternal transmission also has a notable effect on the results of high genetic mutation. 10% paternal transmission under non-Common affinity schemes is sufficient to switch patterns of shared song dominance back to that of the original exclusive songs *A*1 and *a*6 (similar to Fig. 4H*,P*). Paternal transmission is less effective in promoting exclusive songs in Area III. At a 30% transmission rate, only the Group scheme can offset the reduction in exclusive songs produced by all measured mutation levels, and even then there is a promotion of the novel *A*2 and *a*5 phenogenotypes over the original *A*1 and *a*6 phenogenotypes.

### Random Mating

In the preceding simulations, both movement and female mate selection were governed by the same affinity scheme (Figure 1). Here we divorce the two behavioral rules to combine affinity-based movement (*s*>0) with random mating within the genetic predisposition (*s* = 0, the Common scheme). One of the important findings in these simulations was that paternal transmission had no effect on the outcome of the model under random mating. Model results draw from the results above of both the Common scheme and the scheme under which movement operates; however, the combination can occasionally produce patterns not observed under either primary scheme. In general, exclusive song tends to occur less often with random mating. This could be a consequence of the fact sexual selection in this model is frequency dependent, and thus increases the frequency of excusive songs when they are common.

### Spatial Variations (Figs. 6I–L)

**Figure 6:**
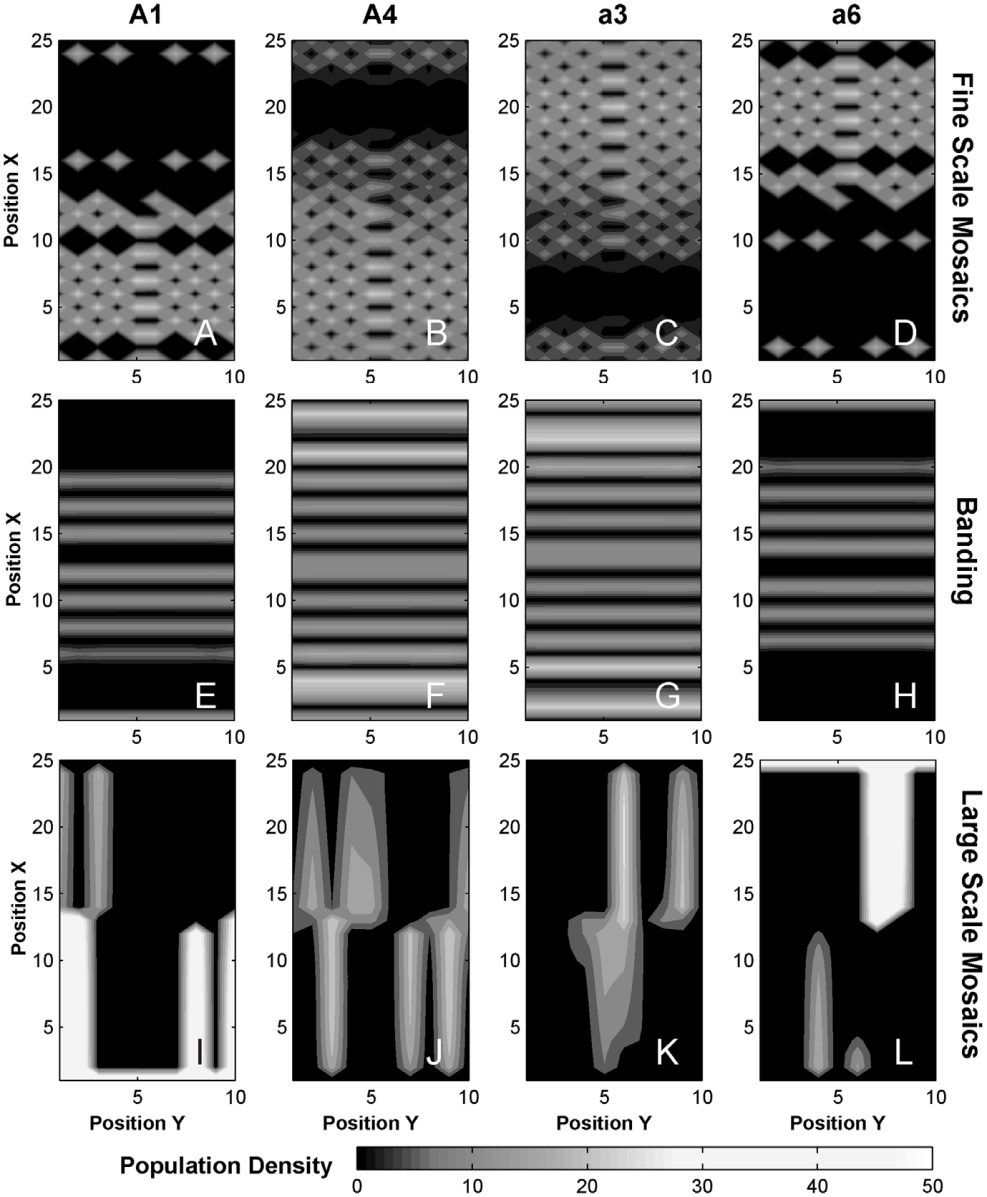
Mosaics and bands. Representative distributions of phenogenotypes *A*1, *A*3, *a*4, and *a*6 are shown as contour plots across the grid of patches (x,y) for fine scale mosaics (A–D, Area II, Match), bands (E–H, Area IV, Common), and large scale mosaics (I–L, Area III, Match). For the large scale mosaics, the initial densities for *A*1 (*x = 1*) and *a*6 (*x = 25*) was drawn from a Gaussian distribution of mean 10 and standard deviation 2. Diffusion and paternal transmission were set to 0.

By varying the initial density of *A*1 and/or *a*6 along their initial invasion edges, we often obtained continuous clusters or large scale mosaic patterns of specific phenogenotypes occurring at a broad scale (“blocks” rather than isolated patches in the fine scale mosaics [Fig. 6A–D] or bands [Fig. 6E–H]). This occurrence was not a replacement of the previously mentioned fine scale mosaic pattern (Area II), but rather it was triggered predominantly when movement by song affiliation and movement by resource were roughly on the same order of magnitude (Area III).

## Discussion

We have examined patterns of song distribution in a zone of secondary contact between two vocally communicating populations, here represented by genetically distinct bird populations with partially overlapping songs or calls. We find several conditions under which population-specific, or exclusive, songs can be maintained in a contact zone; however the maintenance of exclusive songs is by no means universal. We also discuss those features that promote the spread of mixed songs.

When we speak of the maintenance of exclusive songs, we refer to two primary outcomes. In the first case, exclusive songs are locally maintained at patch-sites within the contact zone at roughly equal levels to shared songs (e.g. Figure 3B). In the second case, exclusive songs are locally predominant (e.g. Figure 3E or 4A). In both cases, two key factors seem to bias populations towards higher degrees of exclusive song. The first of these is non-random mating in which females bias their choice of partners within the set of songs that they recognize (e.g., our Group, Match, or Shift affinity schemes, but not the Common scheme). This supports early suggestions that female preference may be important for maintenance of song in the case of dialects (e.g., [20], [24]–[32] but see [33], [34]), favoring dialect maintenance. Next, the maintenance of exclusive song is particularly promoted when there are innate preferences of females for songs that are exclusive to their population (our Group scheme, or songs 1 and 2 under our Shift scheme) rather than when specific song preferences are learned (our Match scheme). Indeed, learning that is not based on population identity can work against the maintenance of exclusive songs because it is a neutral process – it can just as easily reinforce a predominance of shared songs. The final factor that tends to increase the frequency of exclusive song is paternal transmission of song recognition. Evidence suggests this is rare in nature (but see [21], [69] for paternal transmission of song and e.g., [70]–[72] for paternally imprinted mating preferences), but our model demonstrates that even small levels of paternal transmission could have a noticeable effect on increasing the local frequency of population-specific songs when combined with appropriate song templates.

In contrast to those features that promote maintenance of song variation are cultural and genetic mutations, diffusion, and oblique transmission. Song or cultural mutation within a genotype will reduce high frequencies of exclusive songs; however this process also has a natural equilibrium in which all songs are maintained in equal frequencies. It can thus, serve as a rescue mechanism for exclusive songs when they would otherwise be reduced in frequency or eliminated from the population. Increases in the frequency of shared song due to cultural or genetic mutation can be augmented by oblique cultural transmission. Under oblique transmission, songs are learned in proportion to their local frequency, and shared songs, which can be produced by both genotypes, consequently appear as a model to learners of either genotype comparatively more often than if sampling were solely from within the same genetic population. We also observed that diffusion typically led to an expansion of the central interface region in which shared songs dominate, while genetic mutation had a more global effect.

An additional consideration in our model is how the balance between resource availability and song similarity during dispersal affects the maintenance of exclusive songs. Early thinking on dialect maintenance was dominated by the idea that birds may use the presence of similar songs as a cue in choosing territories during dispersal (e.g., [5], [39]). More recent studies instead have favored alternative hypotheses, such as post-dispersal learning (e.g. [35]) or selective attrition of song (e.g., [38]). We find that when socio-cultural factors and resource availability are comparable considerations during dispersal, shared song tends to become more frequent. Singers of shared songs are more likely to disperse from the initial colonization site further into the center of the zone of contact because they are locally socially unfit and/or perceive a given home location as less desirable. The same holds true for phenogenotypes arising from genetic mutation. Inversions, banding, and mosaics are all specific instances of this phenomenon: the colonization front in our model tends to consist of rarer phenogenotypes. In the latter cases, the genotypes tend to macroscopically mix more often than cases in which there are large blocks of similar phenogenotypes. Even in the face of diffusion, fine scale mosaics persist at the highest levels of cultural sensitivity and permit the persistence of both exclusive and shared songs in local enclaves. Mosaic patterns of song variation are found in natural populations (e.g. [6], [39], [73]), but these are single populations, and the causes in these instances are unknown.

In parallel with the findings of our simulations, empirical studies of contact zones and hybrid zones between bird populations demonstrate different patterns of dialect maintenance. A study of flight calls between Australian ringneck parrots (*Platycercus zonarius*) in two contact zones, for example, finds that the calls of one or the other parental subspecies generally dominates in each zone [74], [75]. Similar results have been found across hybrid zones in chickadees (*Poecile atricapillus* and *P. carolinesis*, [76]). In contrast, pied flycatchers (*Ficedula hypoleuca*) have been known to converge towards collared flycatchers (*F. albicolis)* through the development of mixed song, while the latter subsequently diverged to maintain song differentiation [24]. For the warblers *Hippolais polyglotta* and *H. icterina*, too, song was found to converge in a hybrid zone, although different song parameters converged in each species [77]. More data would have to be gathered on a wide variety of hybrid zones before it is known with confidence whether this variety in the consequences of secondary contact is a general finding, or whether one result will emerge as predominant.

The maintenance of population specific song introduces the possibility of further divergence of the populations based on song type, not analyzed in this model. This divergence could occur either if the song types diverge in phenotypic space from one another, or if mating preferences evolve to become so strongly based on song type that they curtail gene flow between the emergent populations. If other sources of selection, such as the low fitness of hybrids, were present in the system it is possible that song may become the basis of divergence during reinforcement, even when song is primarily learned [78]. It is also possible, however, that no further divergence would occur between populations even if song variation were maintained. Irwin [22], for example, did not find reproductive character displacement in song after secondary contact in a ring species of *Phyloscopus* warblers. This species, however, did not interbreed in the contact zone, so hybrids were not created, removing a possible source of selection for divergence (i.e. via reinforcement).

One intriguing aspect to this study is that its results suggest a natural directional bias in the evolution of signaling systems. Our Shift affinity scheme is so named because the “window” of songs recognized by a template, and their associated preference weights, are shifted between the populations. We found that populations that shifted their templates in the direction of increased preference for novel songs over those commonly produced by the larger community of singers were able to establish themselves in the range of a population favoring those common songs. This does not necessitate the loss of the shared songs, nor does it require the elimination of local genetic diversity in such areas, as demonstrated by our simulations with genetic mutation. In an evolutionary context, we could extrapolate that mutants within a communicating population will tend to be successful if they diminish their innate preference of the pre-existing signals or songs and correspondingly elevate their preference for a novel signal or song; however, this hypothesis warrants further examination in the broader class of communication systems.

There are several additional directions in which this research could be further extended. One important question is under which conditions can unbiased preferences give way to biased ones (i.e. evolution of the parameter *s*, thereby moving from a Common scheme to a Group or Match scheme). Similarly, one could ask if there is any evolutionary benefit in using an innately biased scheme (e.g. Group) over a learned one (Match). Moreover, the assumption of a template for song recognition could be modified to consider separate loci controlling song production in males and mating preferences in females with each locus under selection. The fitness of individuals outside of mating could also be incorporated into a more detailed model. For example, there is evidence that males with foreign songs may disperse but have low fitness in their new environment (e.g., [43], [49], [73], [79]), and the potential low fitness of hybrids could become the basis of divergence during reinforcement, even when song is primarily learned [78]. Additionally, there could be some habitat-dependent fitness linked to the genetic basis controlling the predisposition for different song types. Finally, although our model is deterministic, corresponding natural patches my have small population sizes and be more strongly subject to stochasticity. The patterns found in our model should thus be considered expectations around which considerable noise may be found. Exploring these outcomes explicitly would be an interesting future direction.

Other additional features that could be included in the model are based on the role of movement within the contact zone, e.g. we might permit adults to be non-sedentary and capable of additional movement during subsequent dispersal phase, or define the resource landscape as heterogeneous in quality, both factors that would affect the spatial distribution of phenogenotypes (c.f.initial spatial variations led to broad clustering of phenotypes). More importantly, the role of cultural composition in dispersal could be scale-dependent, e.g. high diffusion and cultural affinity could prevail at the micro-scale but resource considerations and diffusion could prevail at the macro-scale. Finally, males of many species produce not one but multiple songs from their repertoire, and the scope of these repertoires may have some effect on the maintenance of more exclusive songs or calls.

In conclusion, the model in this paper demonstrates that many different patterns of song distribution are possible when populations signing different regiolects come into secondary contact. Female preferences for songs common in their natal area – particularly innate ones – can favor the maintenance of population specific songs and discourage the predominance of shared song types, as can paternal transmission or imprinting versus oblique means. To a lesser extent, song-biased dispersal can also support the maintenance of exclusive songs in our model through the formation of small or large-scale mosaics, although we acknowledge the controversy over the existence of such patterns in actual populations. The model we have presented is conceptually extensible, and it suggests similar results for other models of communication involving both genetic and cultural influences.

## Supporting Information

Appendix S1 Simulation Equations.
(DOC)
