# Supplementary material for: Vocal Communications and the Maintenance of Population Specific Songs in a Contact Zone
Source: PLoS One. 2012 May 4;7(5):e35257. doi: 10.1371/journal.pone.0035257 (PMC3344826; doi:10.1371/journal.pone.0035257)
Supplement: Appendix S1 — Simulation Equations. (DOC) [file pone.0035257.s001.doc]

**APPENDIX S1. SIMULATION EQUATIONS**

**For “Vocal Communications and the Maintenance of Population Specific Songs in a Contact Zone” by JT Rowell, MR Servedio**

In this Appendix, we describe the four stages of the life cycle in detail. Numerical results were obtained through the implementation of a Matlab routine available upon request from the authors.

*Step 1: Generation of Offspring Before Song Learning.* Adults within a given patch mate under strict polygyny. Females mate non-randomly with males singing a recognizable song according to song preferences given in Fig. 1 (see also discussion above). All parental combinations are equally fit with regard to the number and viability of offspring, and there is a level of bidirectional genetic mutation (γ_2_) between the *A* and *a* alleles. The adult density for phenogenotype *k* (1≤ *k* ≤ 8) at location (*x*,*y*) is denoted *u_k_*(*x*,*y*), with the phenogenotypes ordered {A1,..., A4, a3,…, a6}. The density of juvenile offspring of genotype *i* (*i* = 1, 2) produced by parents of maternal and paternal phenogenotypes *j* and *k* (1≤ *j*, *k* ≤ 8) equals

|  |  | (1) |
| --- | --- | --- |

Here *V* is a potential function of resources (either *R_0_* or *R*) and represents the expected number of total offspring per female surviving to adulthood per census, and$p_{ijk}(\gamma_{2})$is the genotypic frequency distribution of offspring for the given parental pair of phenotypes, adjusted for a set level of genetic mutation (γ_2_) (e.g. *p_111_*(γ_2_) = 1 - γ_2_ , *p_211_*(γ_2_) = γ_2_). $\tilde{a}_{jm}$is the preference weight a *j*-type female gives to an *m*-type male based upon the song he sings. If females mate non-randomly within recognizable songs, $\tilde{a}_{jm}$is set by the affinity schemes in Figure 1. If females instead mate randomly within recognizable songs, all non-zero $\tilde{a}_{jm}=1$, (*s* = 0).

*Step 2: Song and Song-Preference Learning.* As juveniles progress to young adulthood, a song (or female preference) is acquired through a probabilistic learning process that includes the random selection of a recognizable model, with phenogenotype *t*, to copy and a non-trivial chance of learning error leading to cultural mutation within the set of allowable songs, γ_1_. In the event of cultural mutation, shifts are equi-probable to the left or right, and they are more likely to result in more similar songs (i.e. song k leading to songs k-1 or k+1) than in those less similar (e.g. songs k-2 or k+2). Here we assume those rates are .45 and .05 respectively. The resulting transition probabilities for *i*-genotype offspring developing phenogenotype *l* after observing model phenogenotype *t* are denoted by $m_{itl}(\gamma_{1})$, 1≤ *t*, *l* ≤ 8.

Learning is generally assumed to be oblique (from neighbors), but our model allows for paternal transmission as well. In oblique learning the probability of learning a song is directly proportional to its local relative frequency (*f_ti_*) among valid model singers (indexed by *t*), given the restrictions of the genetic predisposition ($f_{ti}$over the phenogenotypic set *T_i_*, *T_1_ = {1,…,6}*, and *T_2_={3,…,8}*). Singers of songs exclusive to the other allele type are ignored by the listener (for example an A-type juvenile will never learn from an *a*6 model). The local density of young adults of phenogenotype *l*, with corresponding allele *i*, is thus

|  | $Y_{l}(x,y)=\sum_{jk} \sum_{t} m_{itl}(\gamma_{1})f_{ti}(x,y)J_{ijk}(x,y)$ | (2) |
| --- | --- | --- |

For scenarios involving paternal imprinting, if the father sings a valid song to his offspring, paternal learning replaces the model frequency $f_{ti}$with $(1-p)f_{ti}+p\delta_{t,k}$, where *p* is the weight given to paternal transmission and $\delta_{t,k}$ equals 1 whenever *t = k*, and 0 otherwise. In the event that there are no valid models from which to learn, the song or song preference is uniformly drawn from the set of songs allowed by the genetically linked template.

*Step 3: Movement.* After song learning, young adult offspring may relocate to any of the four adjacent patches or remain in their natal patch. Note that only young adults disperse; mature adults are presumed sedentary. Movement occurs based on a weighted combination of three factors: 1) it may be directed towards an increase in per capita resource or space availability with a relative rate set by $\omega_{1}$, 2) it may be directed towards locations that have a strong representation of preferred songs and away from areas where less preferred songs are prevalent with a rate set by $\omega_{2}$, and 3) it may originate from an unbiased random diffusion process among nearest neighbors irrespective of the local cultural or resource landscapes, with a rate set by $\omega_{3}$. Post-movement densities of young adults, $N_{l}$, are thus given by

|  |  | (3) |
| --- | --- | --- |

where *Y* is the density of pre-dispersal young adults computed in (2) whose song or preference has been set, and *x’* and *y’* are standard dummy indices on summations identifying neighboring patch coordinates. Note that the diffusion rate $\omega_{3}$is set to zero as a default. The notation $\left\lfloor F\right\rfloor$ should be understood as a floor function setting negative arguments to 0; i.e. directed individuals will not enter a lesser evaluated patch (for more on directed movement, see [58,59] and references therein).

The resource-based landscape, or search measure, is a simple interference model which is proportional to the amount of available resources or space and inversely related to the population density,

|  | $R(x,y)=\frac{R_{0}}{D+\sum_{i} u_{i}}$. | (4) |
| --- | --- | --- |

The resource measure is identical for all phenogenotypes, but the cultural metric is dependent upon the songs favored (female) or sung (male) by the individual sampling his patch neighborhood. The equivalent cultural landscape, *L_i_*(*x*,*y*), is taken as a linear function of population densities of individuals singing different songs,

|  | $L_{i}(x,y)=\sum_{j} (2a_{ij}-1)u_{j}$. | (5) |
| --- | --- | --- |

The weighting terms are derived from the affinity scores presented in the Fig. 1 such that they range from -1 to 1. There is thus an inherent tendency to actively avoid areas dominated by exotic songs (*a_ij_* < .5) in favor of unpopulated territory.

*Step 4. Mortality.* The newly settled juvenile population adds to the existing local sedentary adult population. The new total adult population then undergoes density dependent per capita mortality,

|  | $\mu=\left(\mu_{0}+\beta_{1}\sum u_{i}\right)/\left(1+\beta_{2}\sum u_{i}\right)$. | (6) |
| --- | --- | --- |

Standard parameter values not reported in the main text are reproductive value, *V(R_0_)* = .4; base mortality, μ_0_=.5, with density-dependent terms β_1_=β_2_=.005; common patch resource or space availability, *R*_0_=10; and interference term, *D*=1.
